# Breast Carcinoma En Cuirasse: Proposal of a Novel Diagnostic Criterion

**DOI:** 10.7759/cureus.116130

**Published:** 2026-09-12

**Authors:** Lyronne Olivier, Karuna S Gopeesingh

**Affiliations:** 1 General Surgery/Breast Surgical Oncology, North West Regional Health Authority, Port of Spain, TTO; 2 Interventional Radiology, Image Guided Solutions Limited, Cunupia, TTO

**Keywords:** armor, breast, breast cancer, breastplate, carcinoma en cuirasse, caribbean health, diagnostic criterion, inflammatory breast cancer, metastatic, palliative

## Abstract

Breast carcinoma en cuirasse (CeC) is a rare form of cutaneous metastases, named as such due to the unique "breastplate" appearance. It is described as diffuse cutaneous induration, thickening, fibrosis, and leathery texture, with erythema and/or hyperpigmentation. Limited literature exists on this rare topic, and subsequently, cases can be misdiagnosed and not appropriately or efficiently managed. As such, we propose the preliminary framework for a novel diagnostic criterion.

This is a case of a 93-year-old woman presenting with a one-year history of progressive left breast swelling, peau d'orange, acute mastalgia, and chest wall and abdominal "breastplate" appearance on examination. No distinct breast masses were palpable, but examination was limited due to the increased skin thickness and leathery texture. Diagnostic breast and abdominal dermal punch biopsies were done urgently at first consultation. Mammogram, breast ultrasound, and computed tomography (CT) were requested.

Histopathology confirmed invasive lobular carcinoma involving the dermis. Immunohistochemistry (IHC) showed positive estrogen receptor (ER) and progesterone receptor (PR). Human epidermal growth factor receptor-2 (HER2) and E-cadherin were negative. Staging CT revealed multiple breast masses, not extending to the skin or chest wall, along with breast and abdominal skin thickening.

Since breast mass biopsies were not performed, we cannot ascertain whether the dermal pathological findings were similar to a primary breast mass. However, since dermal histopathology demonstrated a breast primary and there was CT evidence of left breast masses and no evidence for an alternate source of cutaneous metastases, we can say with fair confidence that the histopathology of the breast masses will be similar to that of the dermal punch biopsies. The diagnosis of breast CeC is therefore supported. Breast CeC contraindicates surgical intervention; thus, definitive medical oncological treatment was implemented. Unfortunately, the patient expired before commencing treatment.

Breast CeC usually develops after initial therapy, and prognosis is typically poor. Differentiating between stage III inflammatory breast cancer (IBC) and breast CeC is crucial, as the management of the former is with curative intent while the latter is with palliation. We introduce the preliminary framework for a novel diagnostic criterion entitled "OG Triple Assessment of Breast Carcinoma En Cuirasse" to elucidate breast CeC and increase diagnostic efficiency. The criterion details conditions that are all required to be present for the proposed diagnosis: (1) clinical appearance of "breastplate"/"armor," (2) no radiological evidence of the direct extension of primary breast tumor to the skin, and (3) histopathological confirmation of carcinoma within the dermis demonstrating morphological and/or immunohistochemical concordance with breast carcinoma.

Breast CeC rarity is evident by its absence in Caribbean-based studies. This being the primary presentation, lobular phenotype, and the only source of metastases made our case even more unique. We aim to introduce breast CeC into Caribbean literature to aid in augmenting pre-existing data. Additionally, establishing future management and diagnostic guidelines, such as the proposed preliminary framework "OG Triple Assessment of Breast Carcinoma En Cuirasse," may aid efficient diagnosis and management and subsequently improve prognosis.

## Introduction

Breast carcinoma en cuirasse (CeC) is a form of cutaneous metastases that is seldom encountered and scarcely documented. From our literature review, there are currently no formally documented cases within the Caribbean. The name arises from the development of the unique "breastplate"/"armor" appearance of the skin [1]. It is most often associated with breast carcinoma and rarely seen as the primary presentation [1]. Differentiation between non-oncological skin thickening, breast cancer skin extension (T4b), inflammatory breast cancer (IBC), and metastatic cutaneous disease from another primary malignancy is imperative. The management of these diagnoses is vastly different, as metastatic therapies are with palliative intent and non-metastatic with curative intent. For example, breast CeC will be managed as stage IV metastatic disease, while inflammatory breast cancer will be managed as stage III non-metastatic disease.

Due to the rarity of breast CeC and limited documentation, minimal guidelines on diagnosis and management exist. Subsequently, it is unknown to many physicians and often misdiagnosed, leading to the late onset of treatment. There are also currently no formally documented cases within the Caribbean in our literature review. As such, we hope to introduce breast CeC into the Caribbean literature and propose the preliminary framework for a novel diagnostic criterion: "OG Triple Assessment of Breast Carcinoma En Cuirasse."

The following report is of an elderly female patient who was referred with left breast pain and peau d'orange, to rule out breast carcinoma. Discussion on diagnosis and management follows.

## Case presentation

A 93-year-old woman presented in November 2023 with a one-year history of left breast swelling increasing in size and a three-week history of ipsilateral breast pain. The peau d'orange appearance was reportedly first noticed earlier in 2023. There was no personal history of breast cancer, and she was unsure of family history.

**Figure 1:**
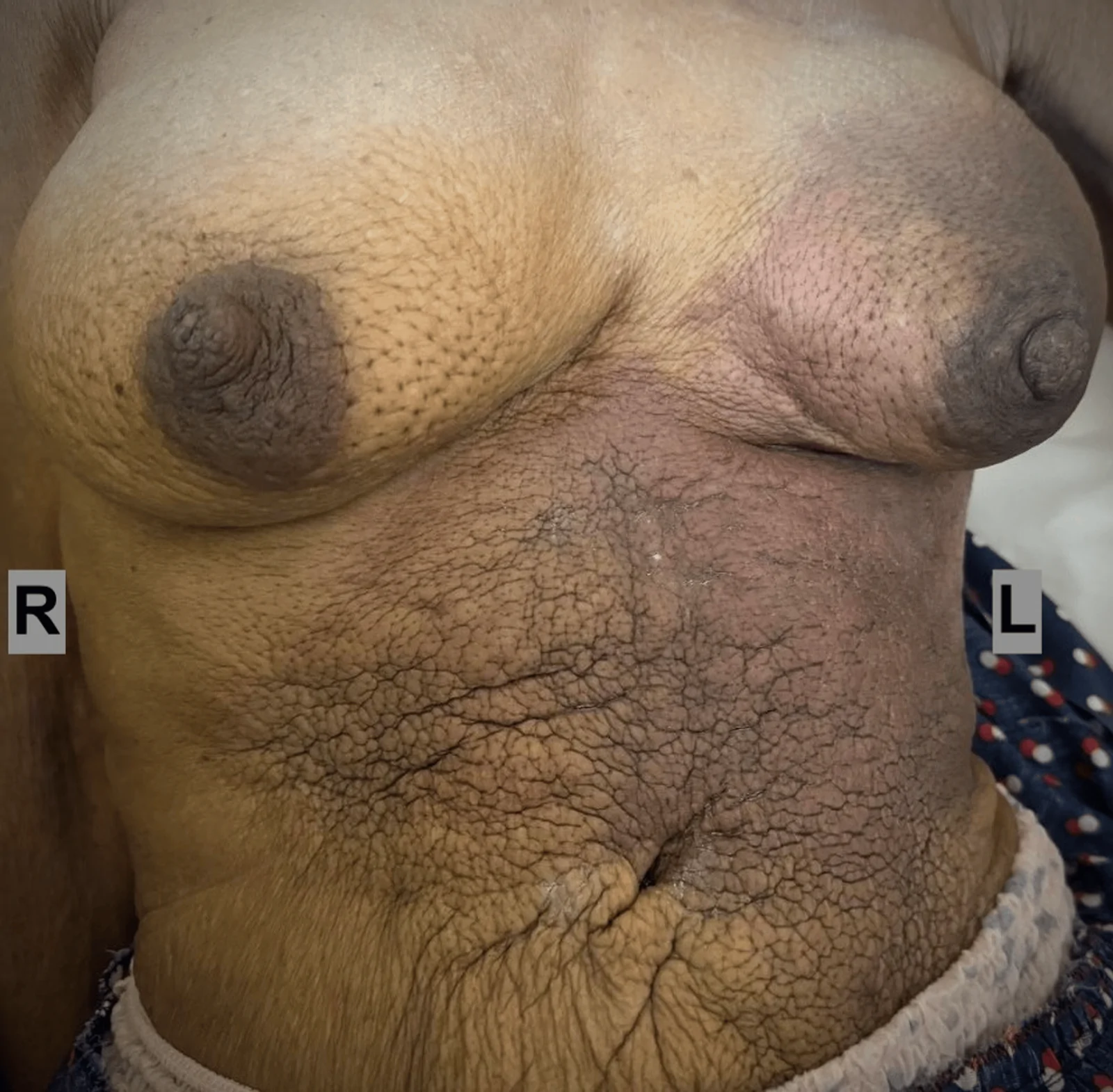
Classic "breastplate" appearance of the anterior left chest wall and abdomen. R, right; L, left

On examination, left breast diffuse leathery infiltrates of the anterior chest wall and abdomen were present (the left more than the right) (Figure 1). Left nipple involvement was also noted, and no nipple discharge was elicited. No distinct breast masses were palpable, but examination was limited because of the extent of the skin thickness and its leathery texture.

The patient was not previously assessed for skin or breast pathologies, and hence, no previous imaging studies or histopathology findings were available for reference.

Due to the apparent advanced disease stage upon initial presentation, the high suspicion for breast CeC, and the limitation with breast palpation, punch biopsies of the breast and abdominal skin were urgently opted for during that first consultation to expedite treatment. Additionally, no breast imaging studies were available on presentation to confirm or exclude the presence of breast masses; therefore, only dermal biopsies were performed. Contrast-enhanced computed tomography (CT) scan of the chest/abdomen/pelvis, mammography, and breast ultrasound were ordered for further assessment and staging. The patient only obtained the CT scan for follow-up review.

**Figure 2:**
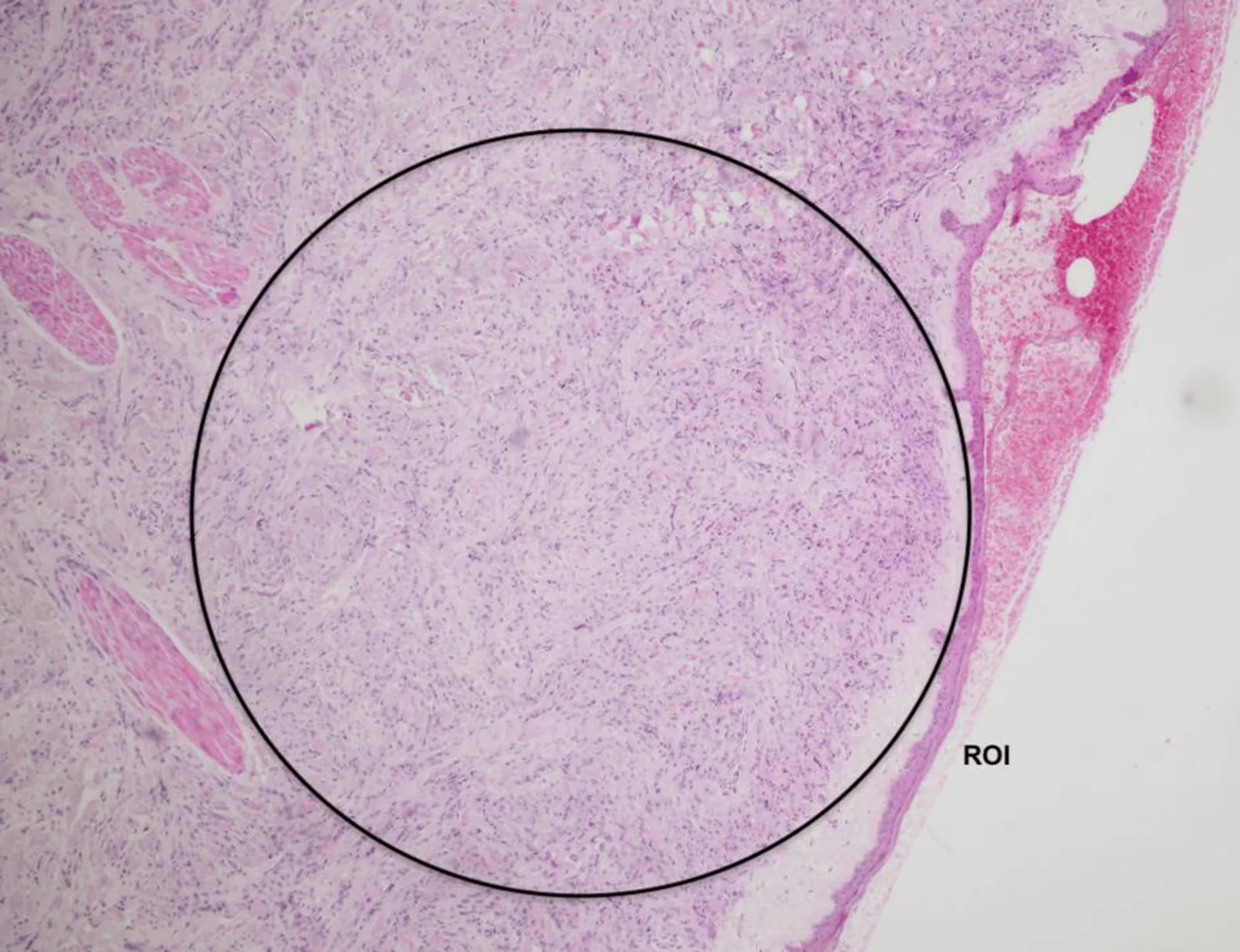
Hematoxylin and eosin (H&E) stain (40×) of invasive malignancy, demonstrating discohesive epithelial cells of the dermis of the skin. ROI: region of interest

**Figure 3:**
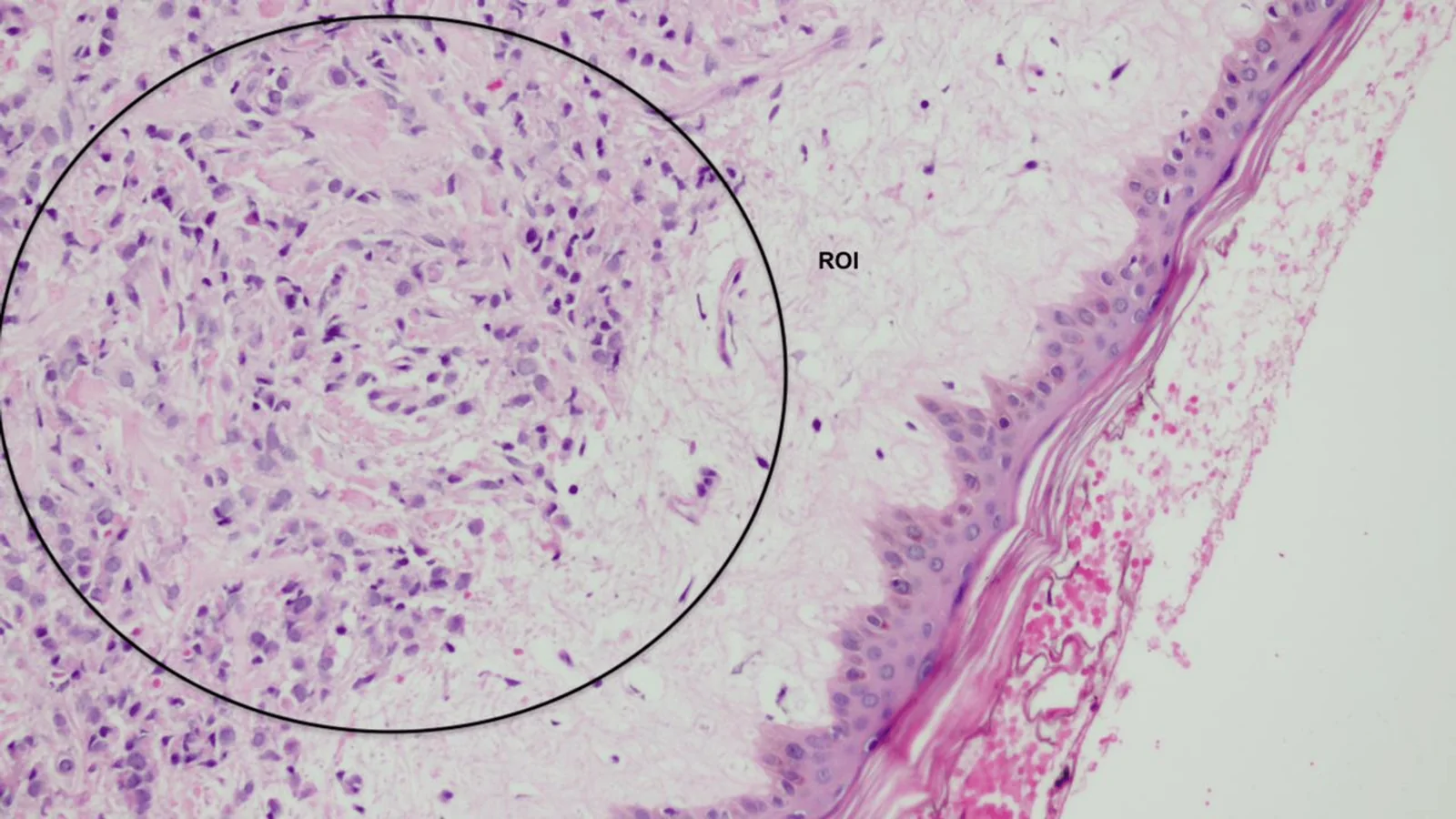
H&E stain as in Figure 2, further magnified (200×). ROI, region of interest; H&E, hematoxylin and eosin

**Figure 4:**
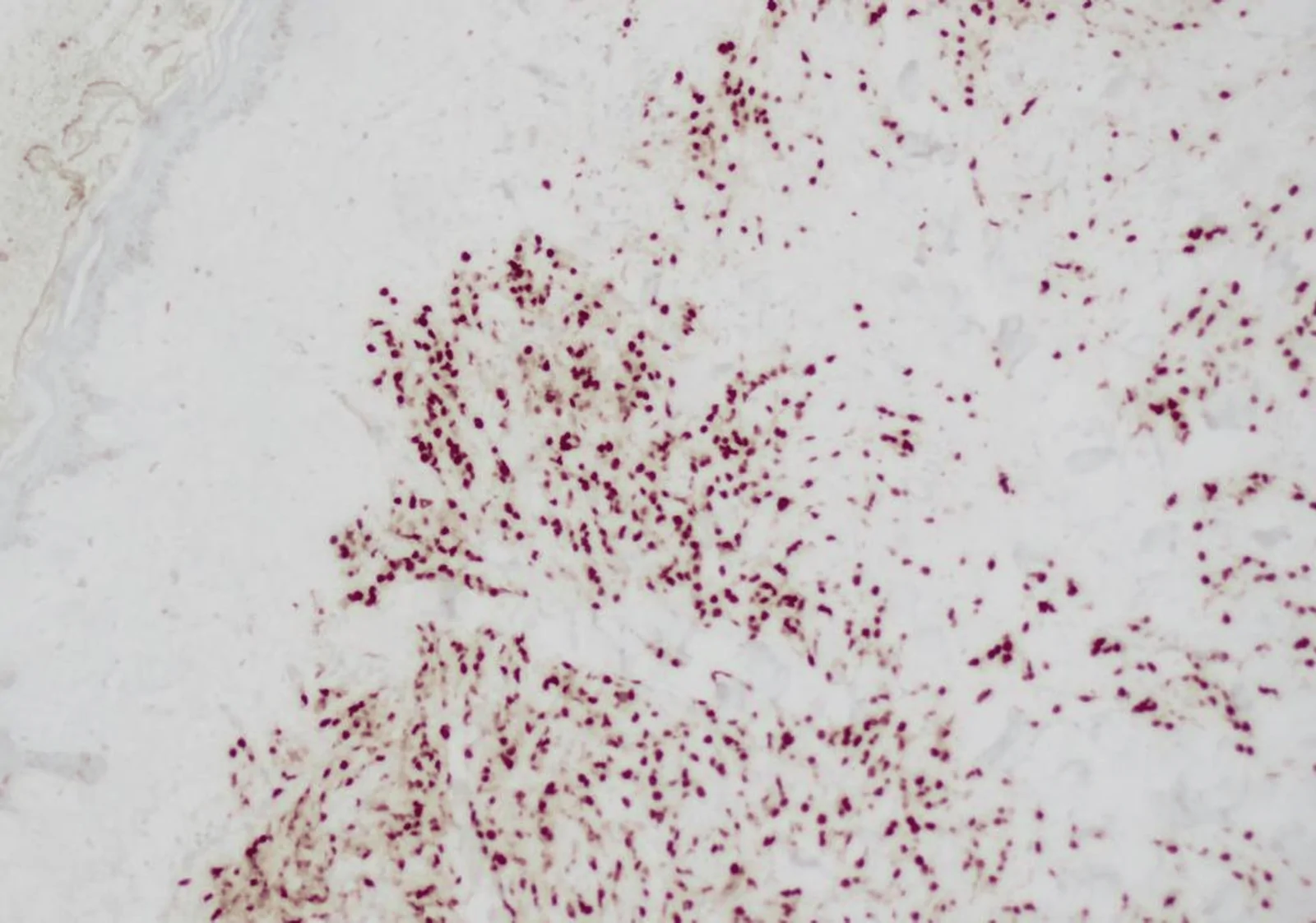
IHC stain (200×) for estrogen receptor highlighting lobular breast carcinoma in the dermis, showing strong nuclear staining. IHC: immunohistochemistry

**Figure 5:**
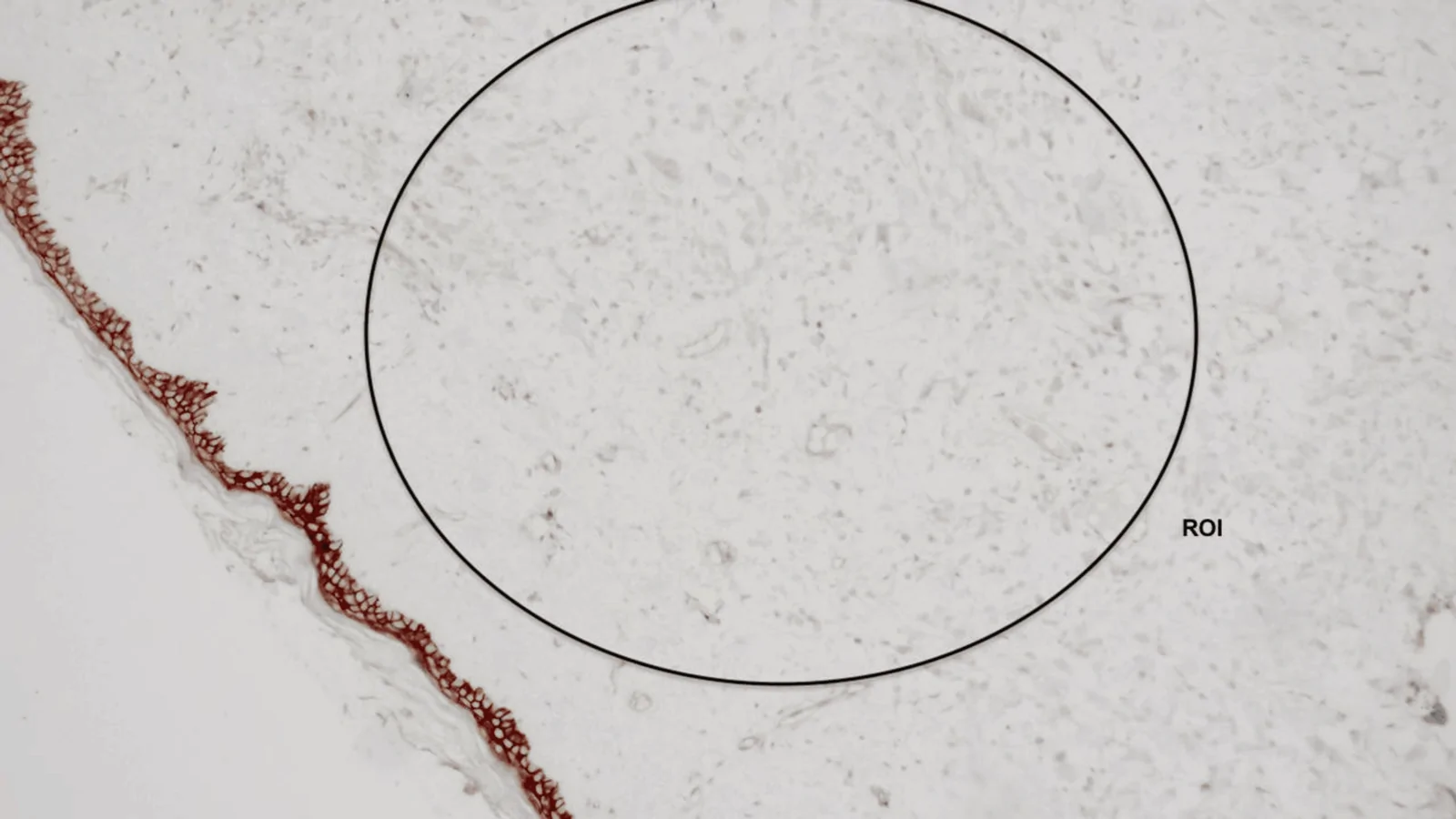
IHC (200×) for E-cadherin demonstrating the lack of staining (E-cadherin loss or E-cadherin-negative). This indicates loss of adhesion, confirming lobular subtype. ROI, region of interest; IHC, immunohistochemistry

Histopathology dated December 2023, addended January 2024, supported invasive lobular carcinoma. Specifically, the left breast punch biopsy reported invasive carcinoma with lobular features involving the dermis of the skin. The left abdominal wall punch biopsy showed metastatic carcinoma involving the dermis of the skin, morphologically compatible with spread from the breast primary. Immunohistochemistry (IHC) showed positive estrogen receptor (ER) and progesterone receptor (PR), with negative human epidermal growth factor receptor-2 (HER2) and E-cadherin. The images below (Figures 2-5) illustrate the key histopathological findings. Images are courtesy of the reporting pathologist, Dr. Melanie Johncilla. 

**Figure 6:**
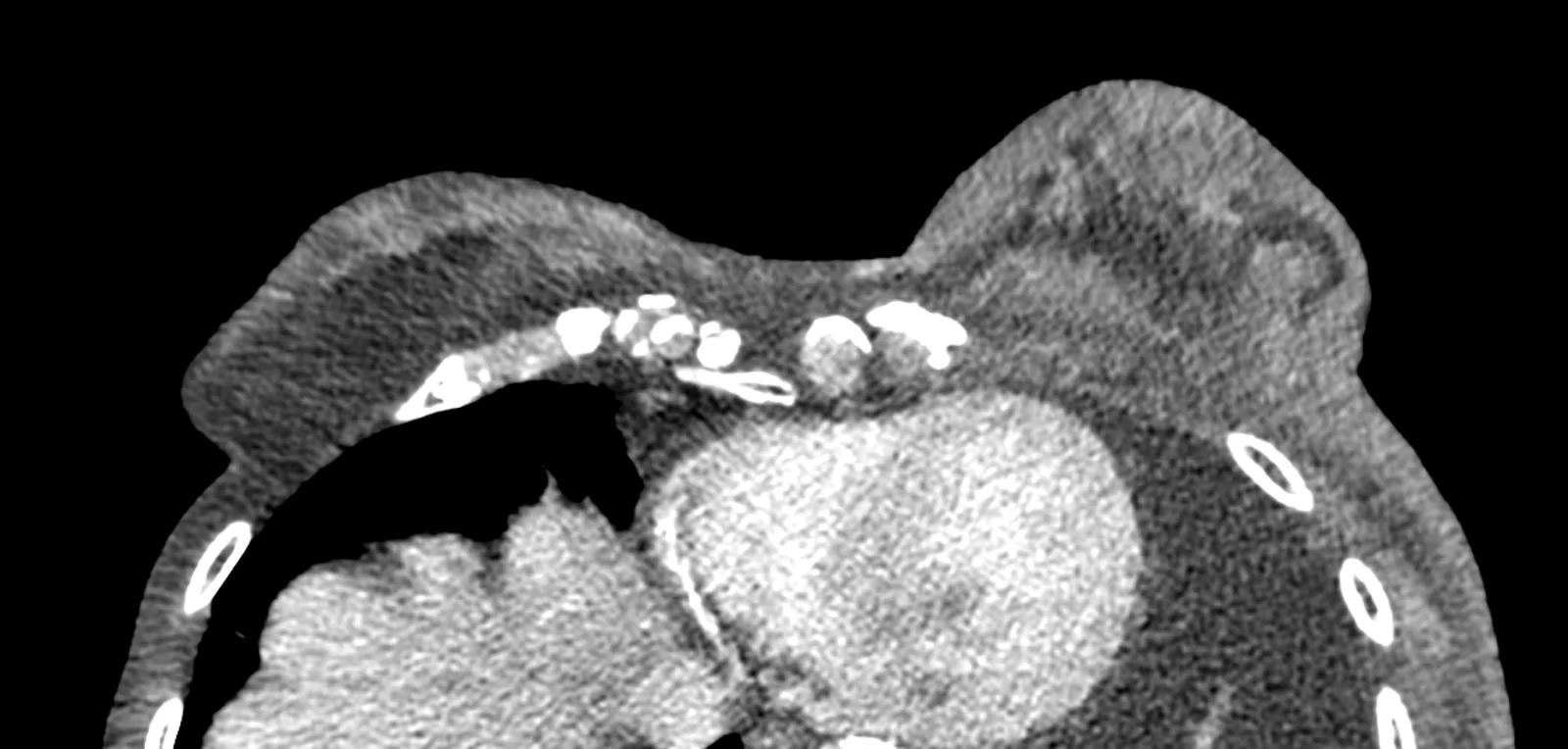
Unlabelled contrast-enhanced CT scan images (partial axial view) of the chest for better appreciation of the abnormalities. CT: computed tomography

**Figure 7:**
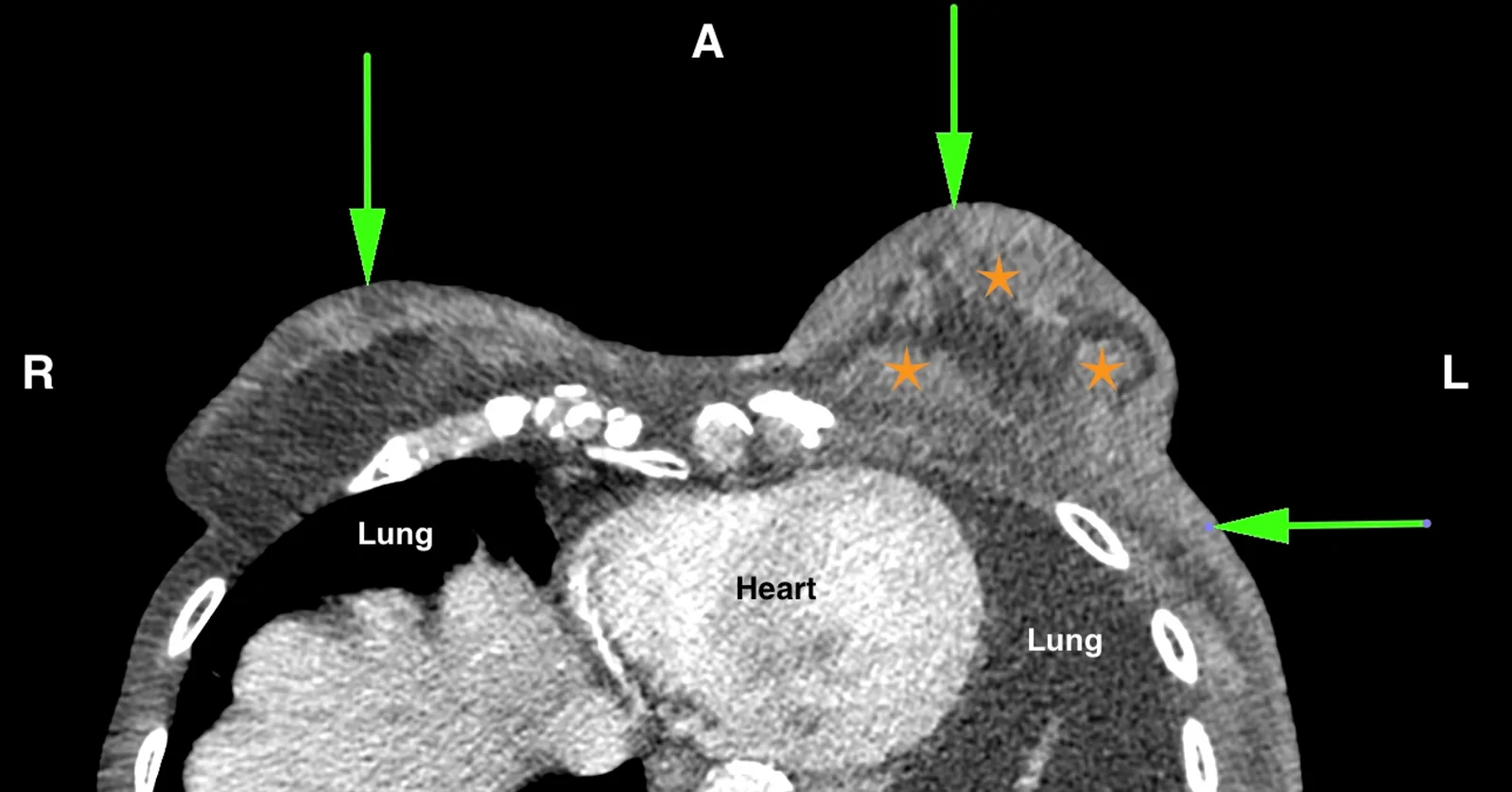
Labelled contrast-enhanced CT scan images (partial axial view) demonstrating irregular skin thickening of the bilateral breasts and anterior chest wall (green arrows). The soft-tissue densities are seen within the left breast (orange star). Also, note a left pleural effusion. The heart and lungs are labelled for reference. R, patient's right; L, patient's left; A, anterior of the patient; CT, computed tomography

**Figure 8:**
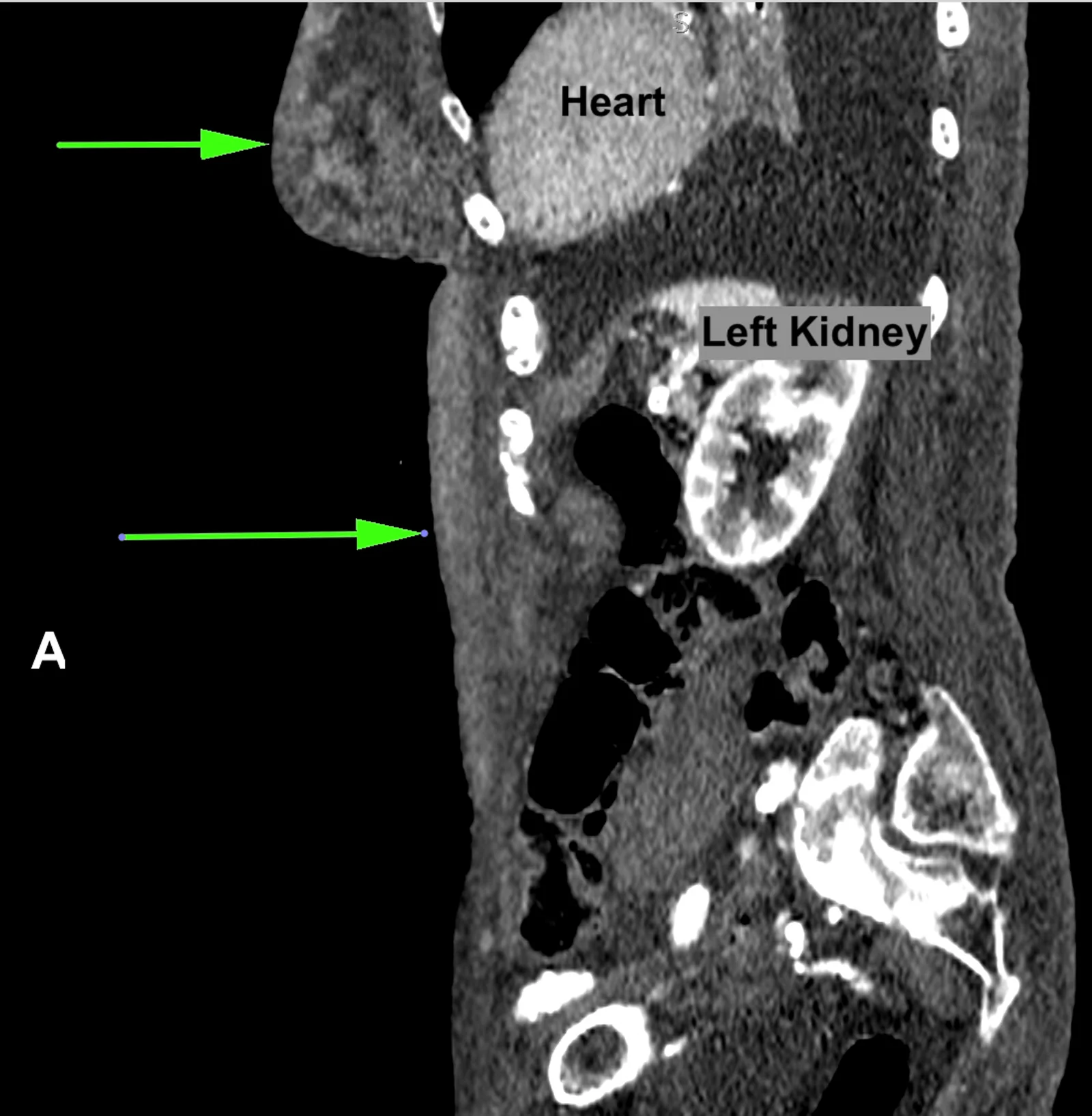
Contrast-enhanced CT scan (sagittal view) of lower chest, abdomen, and upper pelvis. Irregular skin thickening (green arrow) extending from the left breast to the lower abdomen is demonstrated. The soft-tissue densities can also be seen within the visible breast. The heart and left kidney are labelled for reference. A, anterior of the patient; CT, computed tomography

**Figure 9:**
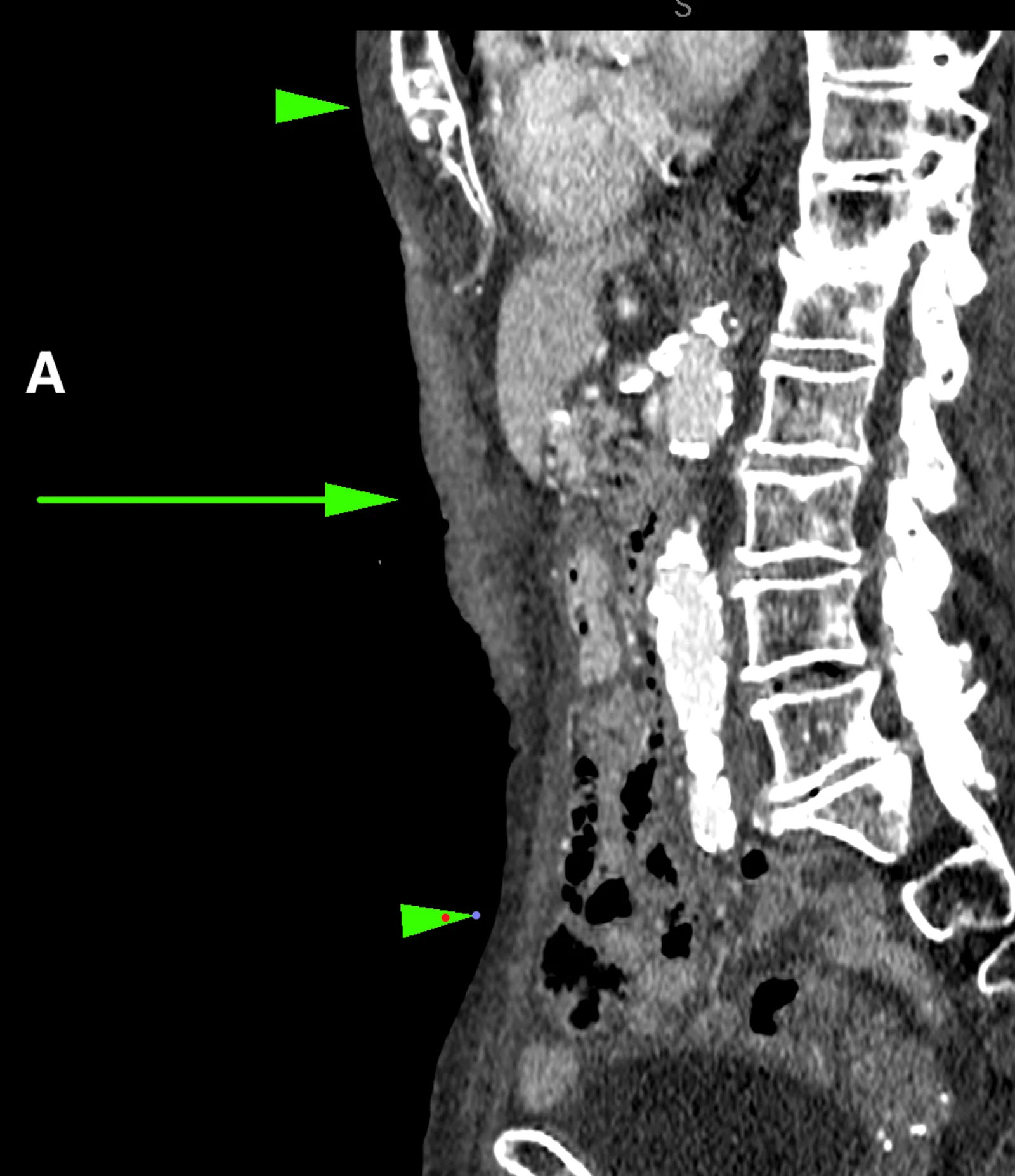
Contrast-enhanced CT scan demonstrating further irregular skin thickening of the abdomen (green arrow). The arrowheads point to areas of no skin thickening. A, anterior of the patient; CT, computed tomography

Contrast-enhanced CT scan of the chest/abdomen/pelvis dated December 2023 (Figures 6-9) demonstrates irregular, bilateral skin thickening of the breast (the left more than the right). Multiple, ill-defined, soft-tissue densities are also present within the left breast with no direct extension to the overlying skin or the underlying chest wall. Skin thickening extended to the left flank and lower abdomen. There were no morphologically suspicious axillary lymph nodes and no evidence of metastatic lesions otherwise. She was subsequently referred to interventional radiology for image-guided breast mass biopsy.

Surgical intervention was contraindicated due to the patient's advanced stage of disease and age on presentation. Our patient unfortunately died in January 2024 before obtaining all her imaging and histopathology results to commence her oncological management.

## Discussion

In 2022, breast cancer had the highest incidence rate of all malignancies in women worldwide. It was also the most prevalent female malignancy in Trinidad and Tobago, with the highest cancer mortality rate in women [2,3]. Mortality is heavily dependent on the stage of presentation [4]. While cutaneous metastases are rare [5,6], approximately 70% of cases are secondary to primary breast cancer according to Brownstein and Helwig [7]. Breast cancer is also the most common female malignancy to metastasize to the skin, accounting for 24% of cases [8,9]. Note that cutaneous metastases are the spread of a primary tumor to the dermis or subcutaneous tissue [10]. Mordenti et al. studied the clinical and histopathological characteristics of 164 cases of skin metastases secondary to breast cancer. Three percent had clinical features of "en cuirasse," and notably, 145 cases had metastases to the trunk [9].

Carcinoma en cuirasse (CeC) is a term used in reference to extensive infiltrating plaques from cutaneous metastasis, involving the skin of the chest with potential spread to adjacent areas such as the neck and abdomen [8]. Typically, it initially appears as nodules that progressively coalesce to form the "breastplate" appearance of the skin, which is diffuse erythema and/or hyperpigmentation with leathery texture and sometimes a nodular and indurated appearance [5,11,12]. CeC is usually associated with breast cancer, with an incidence rate of less than 10%, and is rarely the primary presentation, usually developing after initial therapy, which makes this case of CeC unique [1,5,9]. CeC is usually a sign of advanced disease and poses a poor prognosis [1], with survival documented one to two years after the onset of skin changes [5,8].

Breast carcinoma en cuirasse versus alternate diagnosis

Limited data on the rare condition results in a deficiency of guidelines on diagnosis and treatment. Differentials for breast CeC can include inflammatory breast cancer (IBC), radiation dermatitis and fibrosis, non-oncological skin pathology, and cutaneous metastases from another primary carcinoma [5].

The diagnosis of cutaneous disease can easily be achieved from histopathology confirming the presence of carcinoma involving the dermis. The question however is that if malignancy is present within the dermis of the ipsilateral breast, is it categorized as IBC or CeC/regional cutaneous metastases? It is important to distinguish between stage III breast cancer and metastatic disease to guide management.

The American Joint Committee on Cancer (AJCC) tumor, node, and metastasis (TNM) staging system (eighth edition) describes T4 in general as a direct extension of breast tumor to the chest wall and/or skin. T4b specifically is "ulceration and/or ipsilateral macroscopic satellite nodules and/or edema (including peau d'orange) of the skin that does not meet the criteria for inflammatory breast carcinoma," with T4d being inflammatory carcinoma [13].

While lymphedema secondary to tumor emboli within the dermal lymphatics may be the cause of the skin changes in IBC, histopathological evidence is not required to issue a diagnosis since IBC is fundamentally a clinical diagnosis [14]. Another characteristic of IBC is the quick progression from initial symptoms to diagnosis being a few months. Of note, histologically relevant metastatic deposits to distant organs should measure no less than 0.2 mm [13].

Carcinoma en cuirasse histopathology

While there are no formal histopathological characteristics, ductal phenotype frequently appeared in our literature review, as opposed to lobular observed in our case [1,5,12,15]. In Ronen et al.'s study, 232 cases of breast carcinoma with cutaneous metastases were studied [8]; 126 cases had sole involvement of the skin of the ipsilateral anterior chest, with 106 being confirmed distant cutaneous metastases. Most of the tumors reportedly had histological features of infiltrating ductal carcinoma with similar histological features to the primary, the distant metastases being a more poorly differentiated version. Specific pathological findings were common in a few case reports, particularly tumor infiltration arranged in an "Indian file" pattern [5,6,16] and fibrotic stroma from tumor deposition [5,16].

In our case, the main differentials being considered were IBC; cutaneous metastases secondary to another primary; and breast CeC. After radiological investigation, while we were unable to sample the primary breast lesions, findings of breast masses along with the clinical findings were highly suggestive of a breast primary pathology.

The confirmation of breast carcinoma in the dermis did not clinically fit the appearance, disease progression, or histological specificities for IBC, thereby ruling out IBC. Histopathological findings within the skin pathognomonic for primary breast cancer without clinical or radiological evidence of direct invasion into the skin from an underlying mass, the extensive involvement of the skin of the entire ipsilateral breast, and the unique "breastplate" appearance as previously described all support the diagnosis of CeC-type cutaneous metastases.

Metastases

The histopathology of the abdominal dermis with similar findings as the breast skin tissue further supported the presence of distant metastatic cutaneous disease secondary to a breast primary. According to the American Joint Committee on Cancer (AJCC) TNM classification for breast cancer, eighth edition, histologically relevant metastatic deposits should measure at least 0.2 mm. Additionally, note that breast cancer grading references breast skin specifically [13]. Thus, the involvement of breast carcinoma within non-breast dermis will constitute distant metastases.

While breast CeC in general is referred to as skin metastases, categorization as M1 disease, in particular if only involving regional and ipsilateral breast skin, is unclear since it is not specifically addressed in formal classifications such as AJCC TNM classification [5].

The presence of distant metastatic disease can be radiologically evaluated with contrast-enhanced or positron emission tomography-CT. No evidence of a second primary or further metastases was noted in our case.

Treatment

The treatment of breast CeC will be consistent with managing a stage IV (M1) metastatic breast cancer as opposed to the management of stage III (T4M0) disease. This primarily involves palliation with the reduction of tumor load and the management of symptoms.

In the limited literature on breast CeC, chemotherapy has reported positive outcomes as follows: Eribulin, a microtubule inhibitor, was reported in one study to soften the nodules [5], and another case reported partial response to paclitaxel use, another microtubule inhibitor [17].

Siddiqui and Zaman reported the use of tamoxifen (selective estrogen receptor modulator {endocrine therapy}) in a case of positive estrogen receptor status, showing the improvement of symptoms from as early as one week and the resolution of all skin lesions in 12 weeks [18]. Data has also shown good results with electrochemotherapy [15,16].

Recommendation

**Figure 10:**
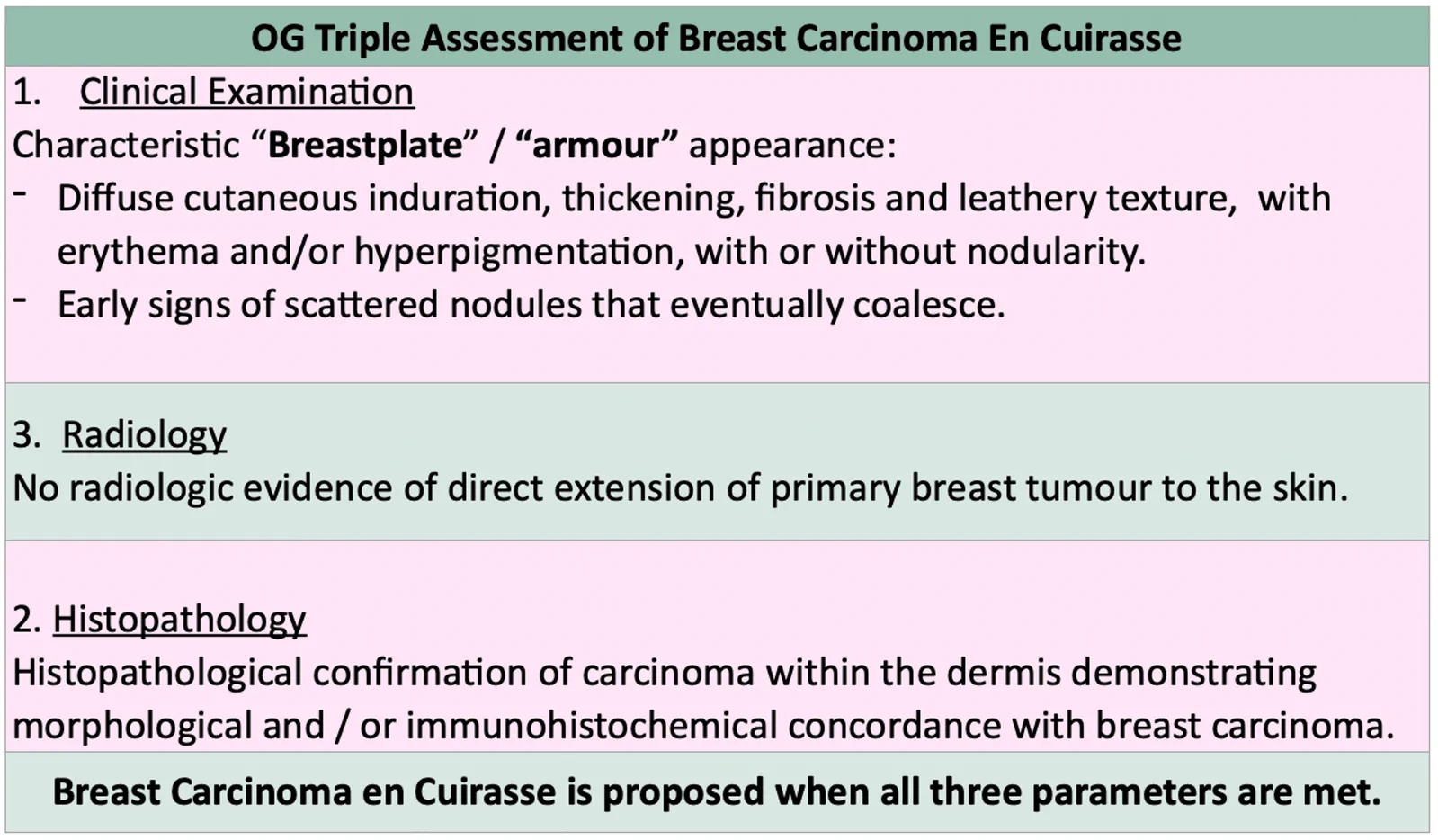
Preliminary framework for proposed novel diagnostic criterion for breast carcinoma en cuirasse.

Differentiating between stage III inflammatory breast cancer and breast cancer (CeC) is crucial for directing management with palliative versus curative intent. We suggest the use of a diagnostic criterion to help elucidate breast CeC and improve diagnostic efficiency. Figure 10 details our proposed preliminary framework entitled "OG Triple Assessment of Breast Carcinoma En Cuirasse."

Our case met the clinical and radiological parameters clearly, with the classic "breastplate" appearance and CT demonstrating the left breast masses with no direct extension to the overlying skin. Histopathology confirmed the presence of breast carcinoma within the dermis even though we were unable to obtain histopathological confirmation from the breast masses specifically. The absolute need for histopathology of the breast masses themselves may not be necessary for management if limitations exist, as in our case. The histopathology of breast masses is particularly warranted in cases requiring oncological treatment or where immunohistochemistry from dermal biopsies was ambiguous.

Limitations

Breast masses were not biopsied at initial consultation because of the increased skin thickness and leathery texture. Though the direct correlation of skin metastasis and breast primary could have impacted clinical management, we cannot ascertain whether the pathological findings of the dermal punch biopsies were in fact similar to the primary breast masses. However, due to the pathological findings being in keeping with a breast primary, CT images demonstrating breast masses, and also the absence of evidence for an alternate source of the cutaneous metastases, we can say with fair confidence that the histopathology of the left breast masses will be similar to that of the dermal punch biopsies.

## Conclusions

In general, metastatic disease usually poses a poor prognosis, and that is no different in the case of breast CeC. Stage III disease management is with curative intent, while metastatic disease undergoes palliative management, hence the importance of raising suspicion for CeC and early distinction between stage III and metastatic disease.

The rarity of breast CeC is evident by its absence in Caribbean-based studies. Breast CeC being the primary presentation, lobular phenotype, and the only source of metastases made this case even more unique. This report aims to introduce breast CeC into Caribbean literature to aid in augmenting the pre-existing data and establishing future diagnostic and management guidelines. Cognizance, clinical suspicion, and the use of diagnostic criteria such as our preliminary framework, "OG Triple Assessment of Breast Carcinoma En Cuirasse," are proposed. This will aid in the efficient diagnosis and management of breast CeC, thereby enhancing overall prognosis.
